# Supplementary material for: Modeling glioblastoma heterogeneity as a dynamic network of cell states
Source: Mol Syst Biol. 2021 Sep 16;17(9):e10105. doi: 10.15252/msb.202010105 (PMC8444284; doi:10.15252/msb.202010105)
Supplement: Supplementary file 6 — Source Data for Figure 5 [file MSB-17-e10105-s004.zip › Figure5A_sourcedata/GSEA_3071/hallmarks_stateIV.GseaPreranked.1623742551702/HALLMARK_E2F_TARGETS.html]

Details for gene set HALLMARK\_E2F\_TARGETS[GSEA]

|  || Dataset | state53071 |
| Phenotype | NoPhenotypeAvailable |
| Upregulated in class | na\_neg |
| GeneSet | HALLMARK\_E2F\_TARGETS |
| Enrichment Score (ES) | -0.5645036 |
| Normalized Enrichment Score (NES) | -3.1430182 |
| Nominal p-value | 0.0 |
| FDR q-value | 0.0 |
| FWER p-Value | 0.0 |
Table: GSEA Results Summary

  

Fig 1: Enrichment plot: HALLMARK\_E2F\_TARGETS      
 Profile of the Running ES Score & Positions of GeneSet Members on the Rank Ordered List

  

| PROBE | GENE SYMBOL | GENE\_TITLE | RANK IN GENE LIST | RANK METRIC SCORE | RUNNING ES | CORE ENRICHMENT || 1 | CDKN1B |  |  | 374 | 0.939 | -0.0634 | No |
| 2 | TFRC |  |  | 1750 | 0.339 | -0.3273 | No |
| 3 | LUC7L3 |  |  | 2124 | 0.280 | -0.3970 | No |
| 4 | ILF3 |  |  | 2159 | 0.274 | -0.4009 | No |
| 5 | LBR |  |  | 2271 | 0.260 | -0.4199 | No |
| 6 | POLD1 |  |  | 2417 | -0.260 | -0.4455 | No |
| 7 | PTTG1 |  |  | 2470 | -0.266 | -0.4530 | No |
| 8 | SHMT1 |  |  | 2478 | -0.267 | -0.4517 | No |
| 9 | LMNB1 |  |  | 2487 | -0.268 | -0.4506 | No |
| 10 | RAD1 |  |  | 2544 | -0.274 | -0.4587 | No |
| 11 | NBN |  |  | 2594 | -0.282 | -0.4655 | No |
| 12 | RNASEH2A |  |  | 2595 | -0.282 | -0.4627 | No |
| 13 | NUP153 |  |  | 2633 | -0.287 | -0.4670 | No |
| 14 | POP7 |  |  | 2651 | -0.290 | -0.4674 | No |
| 15 | CDC25B |  |  | 2671 | -0.292 | -0.4682 | No |
| 16 | XRCC6 |  |  | 2696 | -0.294 | -0.4700 | No |
| 17 | NAP1L1 |  |  | 2698 | -0.294 | -0.4672 | No |
| 18 | CCNE1 |  |  | 2741 | -0.299 | -0.4724 | No |
| 19 | CDC25A |  |  | 2753 | -0.301 | -0.4716 | No |
| 20 | PRKDC |  |  | 2815 | -0.307 | -0.4804 | No |
| 21 | SMC6 |  |  | 2847 | -0.312 | -0.4833 | No |
| 22 | DCK |  |  | 2907 | -0.319 | -0.4916 | No |
| 23 | EZH2 |  |  | 2946 | -0.324 | -0.4958 | No |
| 24 | SMC1A |  |  | 2968 | -0.328 | -0.4966 | No |
| 25 | IPO7 |  |  | 2974 | -0.329 | -0.4943 | No |
| 26 | UBR7 |  |  | 2985 | -0.331 | -0.4930 | No |
| 27 | SMC4 |  |  | 3035 | -0.337 | -0.4991 | No |
| 28 | NUP205 |  |  | 3125 | -0.350 | -0.5130 | No |
| 29 | RPA1 |  |  | 3127 | -0.350 | -0.5097 | No |
| 30 | CDKN2A |  |  | 3146 | -0.353 | -0.5097 | No |
| 31 | MCM2 |  |  | 3240 | -0.366 | -0.5241 | No |
| 32 | MXD3 |  |  | 3316 | -0.377 | -0.5349 | No |
| 33 | POLE |  |  | 3329 | -0.379 | -0.5335 | No |
| 34 | NUP107 |  |  | 3342 | -0.380 | -0.5321 | No |
| 35 | CHEK2 |  |  | 3396 | -0.390 | -0.5385 | No |
| 36 | DCTPP1 |  |  | 3414 | -0.393 | -0.5379 | No |
| 37 | RAD51C |  |  | 3415 | -0.393 | -0.5340 | No |
| 38 | AK2 |  |  | 3461 | -0.402 | -0.5387 | No |
| 39 | TBRG4 |  |  | 3475 | -0.405 | -0.5372 | No |
| 40 | ZW10 |  |  | 3544 | -0.418 | -0.5463 | No |
| 41 | NME1 |  |  | 3548 | -0.419 | -0.5427 | No |
| 42 | CIT |  |  | 3554 | -0.419 | -0.5395 | No |
| 43 | TIPIN |  |  | 3559 | -0.420 | -0.5361 | No |
| 44 | PAICS |  |  | 3594 | -0.427 | -0.5385 | No |
| 45 | EED |  |  | 3708 | -0.447 | -0.5560 | No |
| 46 | NOLC1 |  |  | 3710 | -0.448 | -0.5518 | No |
| 47 | TUBG1 |  |  | 3775 | -0.460 | -0.5597 | Yes |
| 48 | PLK4 |  |  | 3791 | -0.465 | -0.5579 | Yes |
| 49 | KIF18B |  |  | 3807 | -0.468 | -0.5562 | Yes |
| 50 | KIF22 |  |  | 3832 | -0.472 | -0.5562 | Yes |
| 51 | GINS4 |  |  | 3843 | -0.474 | -0.5534 | Yes |
| 52 | DCLRE1B |  |  | 3854 | -0.477 | -0.5506 | Yes |
| 53 | TIMELESS |  |  | 3877 | -0.482 | -0.5501 | Yes |
| 54 | SMC3 |  |  | 3952 | -0.498 | -0.5596 | Yes |
| 55 | DSCC1 |  |  | 3962 | -0.501 | -0.5563 | Yes |
| 56 | POLD2 |  |  | 3964 | -0.502 | -0.5515 | Yes |
| 57 | SLBP |  |  | 3994 | -0.509 | -0.5521 | Yes |
| 58 | NCAPD2 |  |  | 4055 | -0.526 | -0.5585 | Yes |
| 59 | MMS22L |  |  | 4062 | -0.528 | -0.5545 | Yes |
| 60 | TUBB |  |  | 4083 | -0.534 | -0.5530 | Yes |
| 61 | CCNB2 |  |  | 4103 | -0.542 | -0.5514 | Yes |
| 62 | E2F8 |  |  | 4104 | -0.542 | -0.5460 | Yes |
| 63 | SSRP1 |  |  | 4112 | -0.544 | -0.5419 | Yes |
| 64 | SPC24 |  |  | 4194 | -0.565 | -0.5521 | Yes |
| 65 | MRE11 |  |  | 4195 | -0.566 | -0.5464 | Yes |
| 66 | PLK1 |  |  | 4205 | -0.569 | -0.5425 | Yes |
| 67 | HMGB2 |  |  | 4243 | -0.578 | -0.5440 | Yes |
| 68 | MCM6 |  |  | 4273 | -0.590 | -0.5438 | Yes |
| 69 | SPAG5 |  |  | 4290 | -0.595 | -0.5410 | Yes |
| 70 | EXOSC8 |  |  | 4293 | -0.596 | -0.5354 | Yes |
| 71 | ASF1B |  |  | 4328 | -0.606 | -0.5360 | Yes |
| 72 | POLE4 |  |  | 4360 | -0.621 | -0.5359 | Yes |
| 73 | KPNA2 |  |  | 4361 | -0.621 | -0.5297 | Yes |
| 74 | LIG1 |  |  | 4368 | -0.624 | -0.5247 | Yes |
| 75 | POLA2 |  |  | 4379 | -0.627 | -0.5204 | Yes |
| 76 | DNMT1 |  |  | 4388 | -0.632 | -0.5157 | Yes |
| 77 | CSE1L |  |  | 4419 | -0.642 | -0.5151 | Yes |
| 78 | DONSON |  |  | 4444 | -0.652 | -0.5133 | Yes |
| 79 | DDX39A |  |  | 4467 | -0.661 | -0.5110 | Yes |
| 80 | RPA3 |  |  | 4482 | -0.670 | -0.5071 | Yes |
| 81 | BUB1B |  |  | 4483 | -0.670 | -0.5004 | Yes |
| 82 | PA2G4 |  |  | 4497 | -0.678 | -0.4962 | Yes |
| 83 | EIF2S1 |  |  | 4553 | -0.707 | -0.4999 | Yes |
| 84 | NOP56 |  |  | 4556 | -0.708 | -0.4932 | Yes |
| 85 | CTPS1 |  |  | 4572 | -0.718 | -0.4890 | Yes |
| 86 | PRIM2 |  |  | 4585 | -0.728 | -0.4841 | Yes |
| 87 | RFC2 |  |  | 4588 | -0.729 | -0.4772 | Yes |
| 88 | CKS2 |  |  | 4600 | -0.733 | -0.4721 | Yes |
| 89 | BIRC5 |  |  | 4618 | -0.745 | -0.4680 | Yes |
| 90 | RACGAP1 |  |  | 4623 | -0.748 | -0.4613 | Yes |
| 91 | RPA2 |  |  | 4638 | -0.760 | -0.4565 | Yes |
| 92 | DUT |  |  | 4642 | -0.762 | -0.4495 | Yes |
| 93 | USP1 |  |  | 4649 | -0.763 | -0.4431 | Yes |
| 94 | MSH2 |  |  | 4673 | -0.781 | -0.4398 | Yes |
| 95 | MCM7 |  |  | 4704 | -0.800 | -0.4377 | Yes |
| 96 | TCF19 |  |  | 4719 | -0.807 | -0.4324 | Yes |
| 97 | TRIP13 |  |  | 4721 | -0.808 | -0.4246 | Yes |
| 98 | KIF4A |  |  | 4727 | -0.812 | -0.4175 | Yes |
| 99 | RANBP1 |  |  | 4741 | -0.823 | -0.4118 | Yes |
| 100 | RAN |  |  | 4743 | -0.824 | -0.4038 | Yes |
| 101 | CDCA8 |  |  | 4747 | -0.827 | -0.3962 | Yes |
| 102 | POLD3 |  |  | 4752 | -0.830 | -0.3887 | Yes |
| 103 | CENPE |  |  | 4757 | -0.832 | -0.3812 | Yes |
| 104 | MELK |  |  | 4758 | -0.835 | -0.3729 | Yes |
| 105 | CHEK1 |  |  | 4764 | -0.837 | -0.3656 | Yes |
| 106 | TMPO |  |  | 4790 | -0.851 | -0.3620 | Yes |
| 107 | MCM5 |  |  | 4792 | -0.853 | -0.3537 | Yes |
| 108 | LYAR |  |  | 4803 | -0.870 | -0.3470 | Yes |
| 109 | GINS1 |  |  | 4804 | -0.871 | -0.3384 | Yes |
| 110 | CDK4 |  |  | 4810 | -0.874 | -0.3307 | Yes |
| 111 | HELLS |  |  | 4819 | -0.882 | -0.3235 | Yes |
| 112 | MCM3 |  |  | 4834 | -0.901 | -0.3172 | Yes |
| 113 | MKI67 |  |  | 4836 | -0.902 | -0.3085 | Yes |
| 114 | DLGAP5 |  |  | 4857 | -0.922 | -0.3032 | Yes |
| 115 | ORC6 |  |  | 4865 | -0.931 | -0.2953 | Yes |
| 116 | CDCA3 |  |  | 4874 | -0.940 | -0.2875 | Yes |
| 117 | CENPM |  |  | 4881 | -0.947 | -0.2793 | Yes |
| 118 | MYC |  |  | 4883 | -0.947 | -0.2701 | Yes |
| 119 | RFC3 |  |  | 4887 | -0.954 | -0.2612 | Yes |
| 120 | PRDX4 |  |  | 4889 | -0.954 | -0.2519 | Yes |
| 121 | TACC3 |  |  | 4900 | -0.966 | -0.2442 | Yes |
| 122 | AURKA |  |  | 4906 | -0.973 | -0.2355 | Yes |
| 123 | SNRPB |  |  | 4931 | -0.996 | -0.2303 | Yes |
| 124 | PSMC3IP |  |  | 4933 | -0.997 | -0.2206 | Yes |
| 125 | BRCA1 |  |  | 4938 | -1.002 | -0.2114 | Yes |
| 126 | MCM4 |  |  | 4947 | -1.011 | -0.2029 | Yes |
| 127 | ATAD2 |  |  | 4980 | -1.058 | -0.1986 | Yes |
| 128 | HMMR |  |  | 4993 | -1.080 | -0.1902 | Yes |
| 129 | UBE2T |  |  | 5000 | -1.091 | -0.1805 | Yes |
| 130 | MAD2L1 |  |  | 5007 | -1.105 | -0.1707 | Yes |
| 131 | SPC25 |  |  | 5012 | -1.112 | -0.1605 | Yes |
| 132 | AURKB |  |  | 5016 | -1.118 | -0.1499 | Yes |
| 133 | TK1 |  |  | 5020 | -1.126 | -0.1393 | Yes |
| 134 | KIF2C |  |  | 5028 | -1.150 | -0.1293 | Yes |
| 135 | TOP2A |  |  | 5029 | -1.152 | -0.1178 | Yes |
| 136 | MYBL2 |  |  | 5041 | -1.172 | -0.1083 | Yes |
| 137 | DEPDC1 |  |  | 5053 | -1.193 | -0.0986 | Yes |
| 138 | PCNA |  |  | 5060 | -1.214 | -0.0877 | Yes |
| 139 | BRCA2 |  |  | 5069 | -1.222 | -0.0771 | Yes |
| 140 | CDC20 |  |  | 5082 | -1.249 | -0.0670 | Yes |
| 141 | MTHFD2 |  |  | 5101 | -1.297 | -0.0576 | Yes |
| 142 | CKS1B |  |  | 5121 | -1.338 | -0.0480 | Yes |
| 143 | RAD51AP1 |  |  | 5133 | -1.375 | -0.0365 | Yes |
| 144 | DIAPH3 |  |  | 5161 | -1.488 | -0.0270 | Yes |
| 145 | CDK1 |  |  | 5164 | -1.512 | -0.0123 | Yes |
| 146 | HMGA1 |  |  | 5220 | -1.792 | -0.0052 | Yes |
| 147 | RRM2 |  |  | 5232 | -1.875 | 0.0113 | Yes |
Table: GSEA details [plain text format]

  

Fig 2: HALLMARK\_E2F\_TARGETS: Random ES distribution      
 Gene set null distribution of ES for **HALLMARK\_E2F\_TARGETS**
